# Photobiomodulation Suppresses Alpha-Synuclein-Induced Toxicity in an AAV-Based Rat Genetic Model of Parkinson’s Disease

**DOI:** 10.1371/journal.pone.0140880

**Published:** 2015-10-20

**Authors:** Abid Oueslati, Blaise Lovisa, John Perrin, Georges Wagnières, Hubert van den Bergh, Yanik Tardy, Hilal A. Lashuel

**Affiliations:** 1 Laboratory of Molecular and Chemical Biology of Neurodegeneration, Brain Mind Institute, Swiss Federal Institute of Technology (EPFL), CH-1015, Lausanne, Switzerland; 2 Centre de Recherche du Centre Hospitalier de Québec, Axe Neuroscience et Département de Médecine Moléculaire de l'Université Laval, Québec, G1V4G2, Canada; 3 Institute of Chemical Sciences and Engineering, Swiss Federal Institute of Technology (EPFL), CH-1015, Lausanne, Switzerland; 4 Medos International Sàrl, a Johnson&Johnson company, Chemin Blanc 38, CH-2400, Le Locle, Switzerland; 5 Qatar Biomedical Research Institute, Hamad Bin Khalifa University, Qatar Foundation, P.O. Box 5825, Doha, Qatar; University of Nebraska Medical Center, UNITED STATES

## Abstract

Converging lines of evidence indicate that near-infrared light treatment, also known as photobiomodulation (PBM), may exert beneficial effects and protect against cellular toxicity and degeneration in several animal models of human pathologies, including neurodegenerative disorders. In the present study, we report that chronic PMB treatment mitigates dopaminergic loss induced by unilateral overexpression of human α-synuclein (α-syn) in the substantia nigra of an AAV-based rat genetic model of Parkinson’s disease (PD). In this model, daily exposure of both sides of the rat’s head to 808-nm near-infrared light for 28 consecutive days alleviated α-syn-induced motor impairment, as assessed using the cylinder test. This treatment also significantly reduced dopaminergic neuronal loss in the injected substantia nigra and preserved dopaminergic fibers in the ipsilateral striatum. These beneficial effects were sustained for at least 6 weeks after discontinuing the treatment. Together, our data point to PBM as a possible therapeutic strategy for the treatment of PD and other related synucleinopathies.

## Introduction

Parkinson’s disease (PD) is a neurodegenerative disorder characterized by the massive loss of vulnerable neuronal populations in different brain regions, notably the dopaminergic neurons of the substantia nigra *pars compacta* (SNc) [1,2]. In addition to neuronal loss, PD is also characterized by intra-neuronal protein inclusions called Lewy bodies. These inclusions consist primarily of fibrillar and aggregated forms of the presynaptic protein alpha-synuclein (α-syn). Increasing evidence from genetics, animal models and cellular studies suggest that α-syn plays a central role in PD pathogenesis and progression, and other neurodegenerative diseases such as Lewy body disease (LBD) and multiple system atrophy (MSA) [3–5]. Multiplication and missense mutations of the gene coding for α-syn (SNCA) have been linked to early-onset familial forms of PD [6,7]. Therefore, counteracting α-syn-induced toxicity is considered as a viable target for the treatment of PD and related diseases [8,9].

The current PD treatments do not treat the underlying causes of the disease, providing only symptomatic relief [1,10,11], and are associated with debilitating side effects, thereby limiting their effectiveness [1,10,11]. Today, there is general consensus that new PD treatments should move from symptom-alleviating to disease-modifying therapies that aim to stop or at least slow down disease progression [1,11]. During the last decade, there has been increasing interest in exploring the therapeutic potential of near-infrared (NIR) light treatment, also known as photobiomodulation (PBM), for the treatment of several human pathologies, including neurodegenerative diseases (Alzheimer’s disease (AD) and PD), arthritis, ulcers and strokes (8–9). PBM, also called Low Level Light Therapy (LLLT), is defined as the therapeutic delivery of light at low (subthermal) irradiance, typically at specific wavelengths corresponding to molecular adsorptions between 600 and 900 nm. This spectral window also corresponds to a maximum penetration depth in most human soft tissues (9–10). Several studies have reported beneficial effects of PBM by preventing cellular degeneration in several animal models of neurodegeneration [12–14], including toxin-based animal models of PD [15–17] and fly PINK-1 genetic model of PD [18].

In the present study, we investigated the effect of PBM on α-syn-induced toxicity *in vivo*, by assessing the impact of the chronic application of this treatment on motor and cellular impairment, in a rat genetic model of PD. This model is based on unilateral adeno-associated virus (AAV)-mediated overexpression of human α-syn directly in the rat substantia nigra, which results in motor impairment and significant (25–50%) loss of dopaminergic neurons [19,20]. Our results show that daily exposure of the rat model of PD to NIR light for 28 consecutive days could suppress α-syn-induced motor impairment and protect against dopaminergic neuronal loss. Interestingly, this beneficial effect was maintained for several weeks after treatment discontinuation. Collectively, our data point to PBM as a viable therapeutic strategy for the treatment of PD and possibly other related synucleinopathies.

## Materials and Methods

### Plasmid construction and production of recombinant AAV2/6 viral vectors

Human α-syn cDNA was cloned into the AAV-CMV-MCS backbone (Stratagene). The production and titration of the recombinant pseudotyped AAV2/6 vectors (serotype 2 genome/serotype 6 capsid) were performed as previously described [21]. The final titers were 3.7 x 10^9^ transducing units (TU)/ml.

### Stereotaxic injections

*In vivo* study was approved by the Swiss Federal Food Safety and Veterinary Office (Animal authorization n° 2905.2). All surgical and behavioral procedures were performed in accordance with the Swiss legislation and the European Community council directive (86/609/EEC) for the care and use of laboratory animals.

Injections were performed under xylazine/ketamine anesthesia as previously described [22]. Sprague-Dawly female rats (Charles River Laboratories) weighing 180–200 g at the time of surgery were placed in the stereotaxic frame (David Kopf Instruments) and received a unilateral intranigral injection of 2 μl of viral suspension, which corresponds to a viral load of 1.5 x10^7^ TU (transducing units). Injections were performed at a rate of 0.2 μl/min controlled by an automatic pump (CMA Microdialysis). The needle was left in place for an additional 5 min before it was slowly withdrawn. Stereotaxic coordinates for the injections above the substantia nigra *pars compacta* were as follows: anteroposterior (AP): -5.2 mm, lateral (L): -2.0 mm; dorsoventral (DV): -7.8 mm from the skull surface, according to the rat stereotaxic atlas by Paxinos and Watson (1986).

### Treatment with NIR illumination

Rats were illuminated with two 808-nm GaAs laser diodes (RLTMDL-808-2W with PSU-LED power supply (Roithner Lasertechnik, GmbH, Vienna, Austria), coupled to two frontal light diffusers to homogenize the illumination spots (FD1, Medlight SA, Ecublens, Switzerland). The diffusers delivered two spots of about 1 cm^2^ on top of the animal’s head. The animals were illuminated once a day for 100 sec over several weeks. During illumination, awake animals were placed in a dedicated cylinder (40 mm ⌀) to limit their movement and ensure reproducibility of illumination. Sham illumination was performed by placing the animal in the same cylinder with the light turned off. Animals were divided in three groups: two groups were illuminated at two different NIR fluence rates, 2.5 mW/cm^2^ (n = 7) and 5 mW/cm^2^ (n = 7), respectively, and one group was sham-treated (n = 9).

### Measurement of the NIR transmitted to the substantia nigra pars compacta

The light transmission through the scalp and skull was calibrated on a rat head shortly after euthanasia (Table A in S1 File). Measurements were performed using an isotropic probe (IP85, ∅ 0.8mm, Medlight SA, Ecublens, Switzerland) coupled to a photodetector (818-SL) and to the driver (1918-R, Newport Spectraphysics, Darmstadt, Germany). The probe was placed in the direct vicinity of the substantia nigra.

### Behavioral study: the cylinder test

The cylinder test was performed to evaluate the motor impairment induced by α-syn overexpression in the rat midbrain, by quantifying usage deficits of the contralateral forelimb, also called akinesia [23,24]. Briefly, the rats were placed in a 20-cm Plexiglas cylinder and videotaped during their exploratory behavior. A total number of 30 forepaw contacts made on the cylinder wall by the ipsilateral or the contralateral (impaired) forelimbs were scored and the results were expressed as the percentage of impaired forelimb contacts versus total contacts [19,20]. Analysis was performed in a blinded fashion.

### Tissue processing

For immunohistochemistry, the animals were deeply anesthetized and transcardially perfused with 4% paraformaldehyde (PFA; Fluka, Sigma-Aldrich). The brains were removed, post-fixed for 2 h in 4% PFA, and then placed in 25% sucrose for 2 days. Thirty-five-μm-thick coronal sections were cut using a microtome (SM2400; Leica) and the slices were stored at -20°C in cryoprotection solution.

### Immunohistochemistry

Immunohistochemical analysis was performed as described previously [22]. Slices were incubated with the primary antibody anti-tyrosine hydroxylase (1:500; AB152; Millipore) or anti-human α-syn (1:1000; sc-211, Santa Cruz Biotec) and subsequently incubated with biotinylated secondary antibody (1:200; Vector Laboratories) for 3,3'-diaminobenzidine tetrahydrochloride (DAB, Pierce) revelation and Alexa-Fluor secondary antibodies (Invitrogen) for immunofluorescence. Slides were Then mounted on glass coverslips.

Imaging was performed on a Leica DMI 4000 microscope (Leica LAS software) equipped with an Olympus AX70 camera. The confocal acquisitions were obtained on a Zeiss LSM 700 upright confocal microscope (Zen software).

### Unbiased stereological estimation of dopaminergic neurons in the SNc

Dopaminergic neuronal loss was estimated using unbiased stereology according to the optical fractionator principle described by West *et al*., [25]. Briefly, the number of TH-immunoreactive neurons was determined every sixth section (1/6), which represents a total of 9 to 11 coronal sections covering the entire SNc structure. The SNc was delineated at low magnification (20X) and then the dopaminergic neurons were counted under an oil immersion objective (60X). TH+ neurons were counted in a blinded fashion and the results are expressed as the mean ± standard error of the total number of TH+ neuron in the injected side. Analysis was performed using the MBF Stereo Investigator software (version 9.0, MBF Bioscience). The parameters used for the stereological analysis were as follows: grid size, 200 x 180 μm; counting frame, 75 x 75 μm; and 2 μm guard zones. Tissue thickness was determined at each counting field. The coefficient of error was < 0.1.

### Statistical analysis

Statistical analysis for cellular quantifications was performed using one-way ANOVA followed by Tukey’s multiple comparison test. For behavioral analysis, statistics were performed using a two-way factorial ANOVA followed by Bonferroni's multiple comparisons test. *p*<0.05 was required for rejection of the null hypothesis. All values were expressed as mean ± s.e.m. The software used for the statistical analysis was Prism v. 6 (GraphPad, La Jolla, CA, USA).

## Results

### Assessment of the optimal parameters for the photobiomodulation treatment

To investigate the effect of the PBM treatment on α-syn-induced toxicity in the AAV-based rat model of PD, we first sought to determine the optimal light fluence rates and treatment duration that would provide the most beneficial outcomes, without inducing deleterious side effects.

We first calibrated the NIR light fluence rates in the deep brain structures of freshly sacrificed rat, including the substantia nigra. As reported in Table A in S1 File 1, we assessed the light fluence rates in the deep structures of the rat brain and their corresponding values of the light irradiance at the scalp surface. We used this data to determine the light fluence rates delivered to the animals in the different experimental groups, from the irradiance applied to the surface of the rat’s head.

We then generated the rat genetic model of PD by unilaterally injecting AAV particles that overexpress human α-syn in the midbrain [19,20]. After three weeks of recovery (post-injection), awake animals were exposed daily to NIR light (808 nm) at different irradiations, for 100 sec. The treatment consisted of animal exposure to NIR light for two weeks (treatment 1), followed by 6 weeks of treatment withdrawal, to assess the sustainability of the treatment effect, and finally further animal exposure to the treatment for 8 consecutive days (treatment 2) (Fig A in S1 File). Five experimental groups were exposed to different NIR light fluence rates in the midbrain 5 mW/cm^2^ (n = 6), 10 mW/cm^2^ (n = 6), 20 mW/cm^2^ (n = 6), 25 mW/cm^2^ (n = 6) and 30 mW/cm^2^ (n = 6). The control group (n = 5) received AAV injection with sham NIR illumination. Analysis of the rat motor performances, using the cylinder test, revealed that only the low fluence rates (5 and 10 mW/cm^2^) reduced the α-syn-induced akinesia, whereas the animals treated with higher light fluence rates, as well as the non-treated animals, exhibited significant reduction of contralateral use, compared to their pre-injection scores (Fig B in S1 File). At the cellular level, our data showed that the majority of the dopaminergic neurons overexpress human α-syn (Fig C in S1 File). Moreover, none of the treatments significantly affected α-syn-induced dopaminergic neuronal loss in the substantia nigra (Fig D in S1 File) or the dopaminergic fiber denervation in the striatum (Fig E in S1 File). However, the treatment with the lowest fluence rate (5 mw/cm^2^) showed a tendency to mitigate α-syn-induced toxicity (Figs D and E in S1 File).

Based on these results, we decided to pursue the study by re-evaluating the effect at 5 mW/cm^2^, as well as a lower fluence rate (2.5 mW/cm^2^), for a longer duration of treatment (4 weeks), on α-syn-induced motor and cellular degeneration in our rat model of PD.

### Chronic treatment with photobiomodulation suppresses α-syn-induced motor impairment

**Fig 1:**
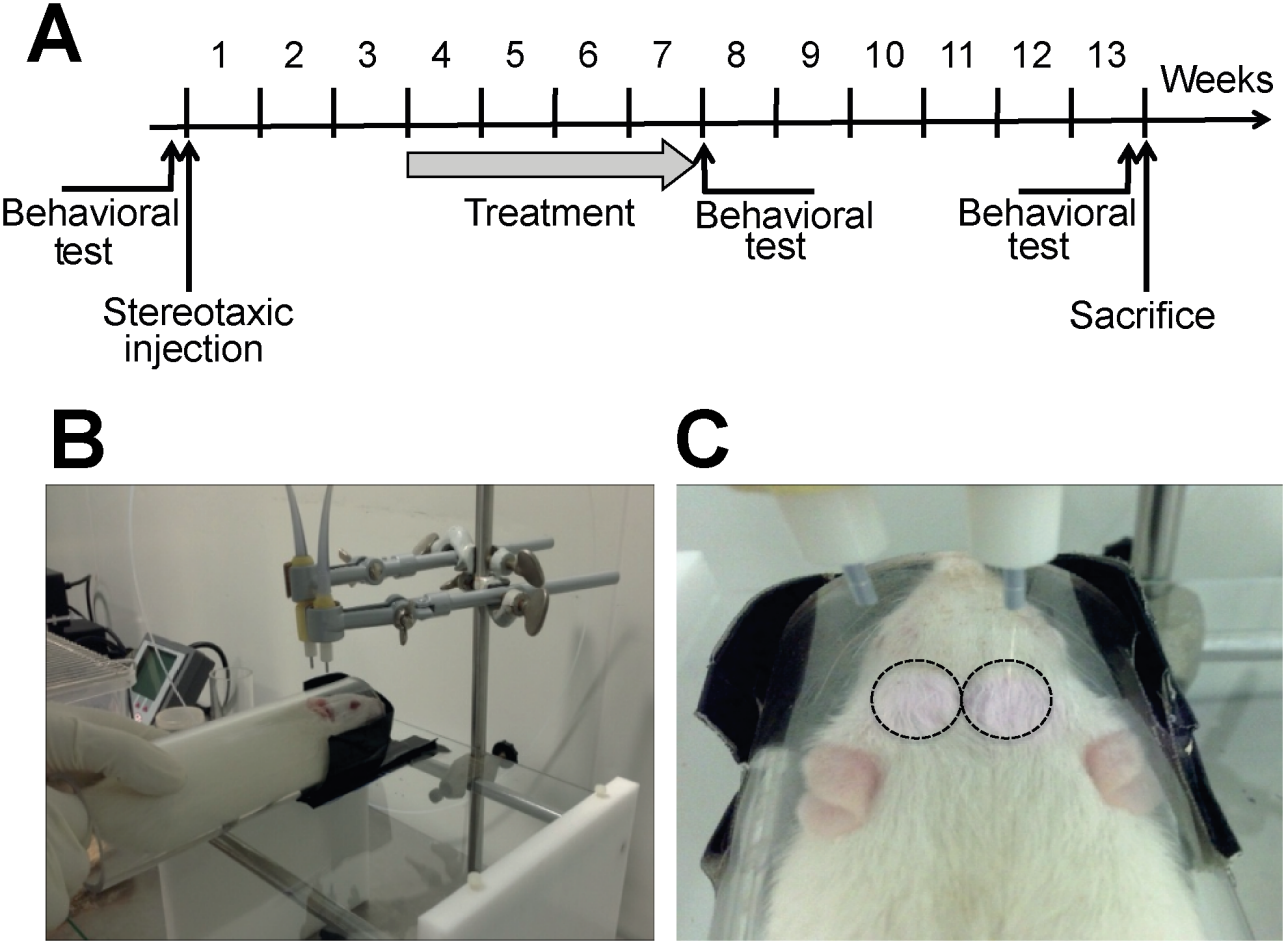
Experimental design. (A) Schematic representation of the experimental timeline. (B) Illustration of the experimental procedure showing how the rats were maintained in a 4-cm transparent Plexiglas cylinder to standardize the distance to the lasers and the effective exposure time for each animal. (C) Illustration of the application of the NIR light using 2 laser sources. The circles delimit the surface of the head exposed to the light, avoiding the animal’s eyes and ears.

In an independent experiment, we evaluated the effect of PBM at low fluence rates on α-syn-induced motor impairment. Three weeks post-viral delivery, rats overexpressing human α-syn were exposed daily to two NIR light beams (808 nm) at different fluence rates (2.5 or 5 mW/cm^2^) for 100 sec. In this experiment, animals were exposed to a chronic PBM treatment for four consecutive weeks and their motor performances were then evaluated using the cylinder test (Fig 1A). To assess whether the PBM effect could be sustained after treatment withdrawal, we maintained the rats without treatment for 6 weeks. Finally, after assessment of their motor performances, the rats were sacrificed and their brains were collected for the cellular analysis (Fig 1A). During PBM treatment, awake animals were temporarily confined in a transparent Plexiglas cylinder (40 mm ⌀) to limit their movement and to maintain their head at a determined distance from the beams. This strategy allowed the delivery of similar pre-determined light doses and reproducible fluence rates to each experimental group (Fig 1B). The light was delivered bilaterally using two frontal light diffusers (Fig 1C). In the sham group, rats were not exposed to NIR light.

**Fig 2:**
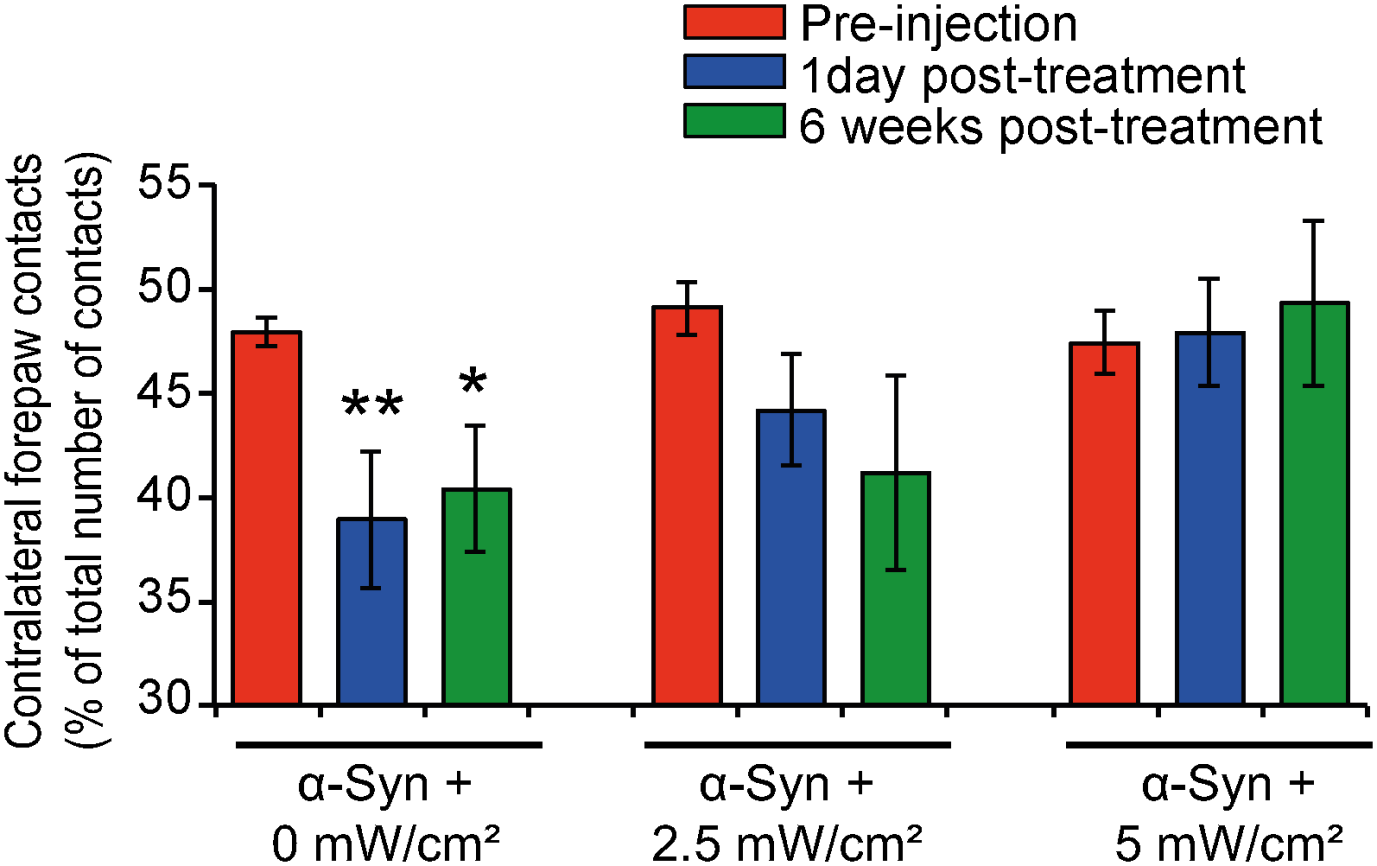
Chronic treatment with photobiomobulation suppresses α-syn-induced motor deficits. α-syn-induced motor deficits were assessed using the cylinder test. The results show that, before injection (red histograms), the animals in all our experimental conditions exhibited essentially symmetrical motor activity, with 50% of use for ipsilateral and contralateral forepaws. The control rats, overexpressing human α-syn without any treatment, exhibited significant reduction of the use of the contralateral forepaw (akinesia), five weeks post-injection (blue histograms) (**p<0.01 *vs*. pre-injection scores, Bonferroni's test). This deficit was also observed 4 months post-injection (-12%) (green histograms), when compared to their pre-injection scores (*p<0.05 *vs*. pre-injection scores, Bonferroni's test). However, the rats exposed to NIR light did not show any motor deficits, when compared to their pre-injection performance, right after the treatment (blue histograms) or 6 weeks after discontinuing the treatment (green histograms).

The rat motor performances were assessed using the cylinder test [23,24], one of the most sensitive behavioral tests to evaluate motor impairment after mild dopaminergic neurons induced by α-syn overexpression [26–28]. This test consists in the evaluation of the utilization of contralateral forepaw, which in the case of α-syn-induced dopaminergic loss, is reduced [19,20]. Before viral injection, the rats use their forepaws equally, with ~50% utilization for each side (red histograms; Fig 2). As we previously showed, overexpression of α-syn induced a significant motor dysfunction in the absence of any treatment (2 months post-injection), as reflected by the reduction in the use of the contralateral forepaw (blue histograms), also referred as akinesia (Fig 2) [19,20]. Interestingly, analysis of the motor performances, a few hours after the last light exposure session, showed that rats treated with PBM at the fluence rates of 2.5 or 5 mW/cm^2^ did not show any significant impairment of their motor performances, compared to their pre-injection scores. This result demonstrates that treatment with PBM, at the fluence rates of 2.5 or 5 mW/cm^2^, was able to counteract α-syn-induced motor deficits.

To investigate whether the beneficial effect of the PBM was sustained after interruption of treatment, we evaluated the rat performances 42 days after the last light exposure. As shown in Fig 2, the treated groups continued to exhibit normal motor performances, as compared to their pre-injection scores, and visibly less akinesia compared to the non-PBM treated group. These observations demonstrate that the beneficial effect of PBM on α-syn-induced akinesia do not require continuous light application and can persist for several weeks after the treatment is discontinued.

### Chronic treatment with photobiomodulation protects the dopaminergic neurons against α-syn-induced toxicity

**Fig 3:**
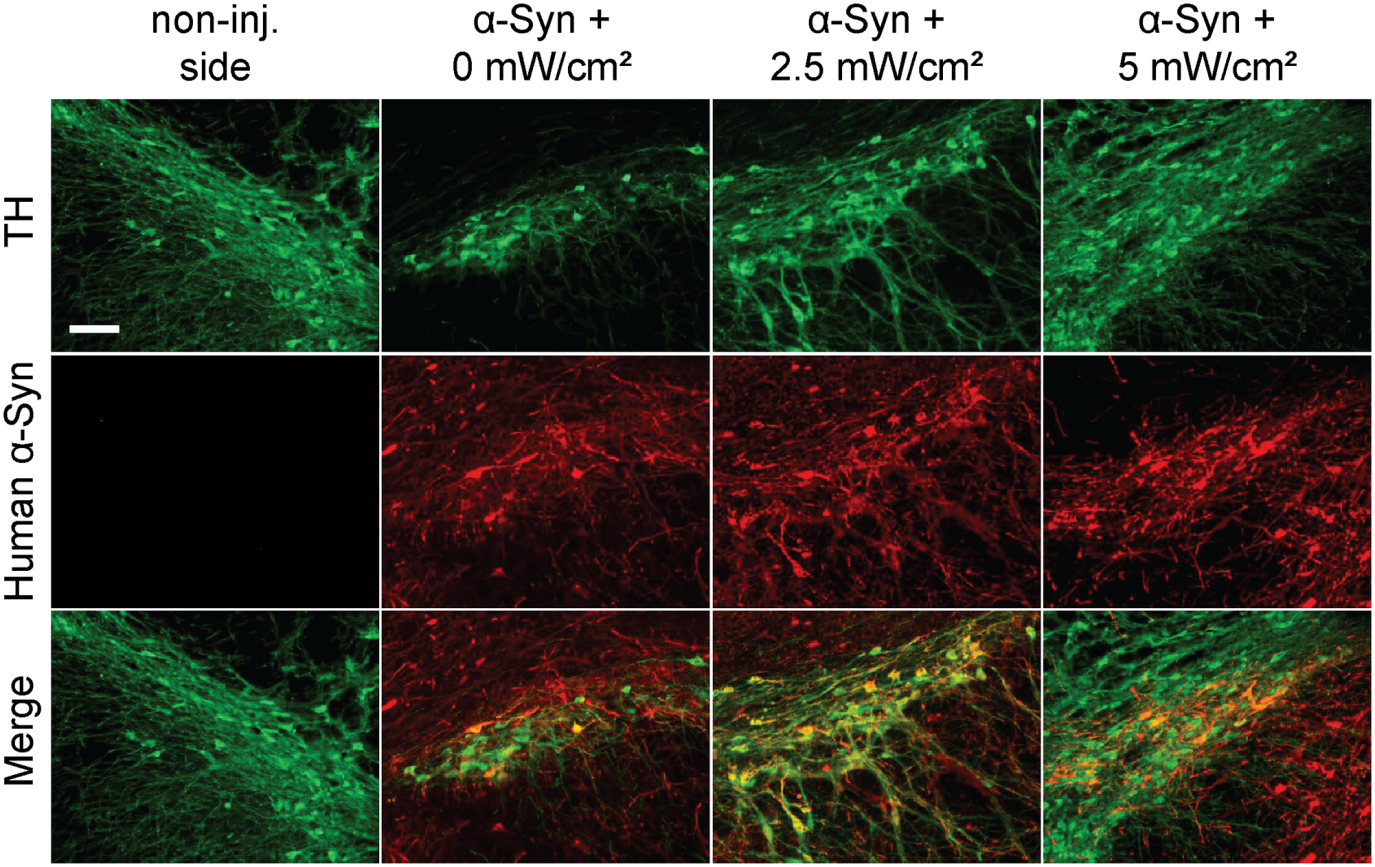
Expression of human α-syn in the injected substantia nigra. Photomicrographs illustrating the detection of human α-syn expression (red) in the nigral TH+ dopaminergic neurons (green). Tissues were sampled after 3 weeks of treatment followed by 6 weeks of treatment withdrawal. The merged images show that human α-syn mainly localizes in the majority of the dopaminergic neurons in the injected midbrains. No human α-syn signal was observed in the non-injected side. No significant difference in the levels or pattern of human α-syn expression between the different experimental conditions was observed. Scale bar = 500 μm.

To investigate, at the cellular level, the effects of PBM on α-syn-induced akinesia, we first confirmed the expression of human α-syn in the injected midbrains. As shown in Fig 3, double immunofluorescence using antibodies against tyrosine hydroxylase (TH), a marker of the dopaminergic neurons, and human α-syn (SC-211) revealed that the majority of the nigral dopaminergic neurons overexpress the exogenous transgene. No human α-syn signal was detected in the non-injected contralateral midbrain (Fig 3).

**Fig 4:**
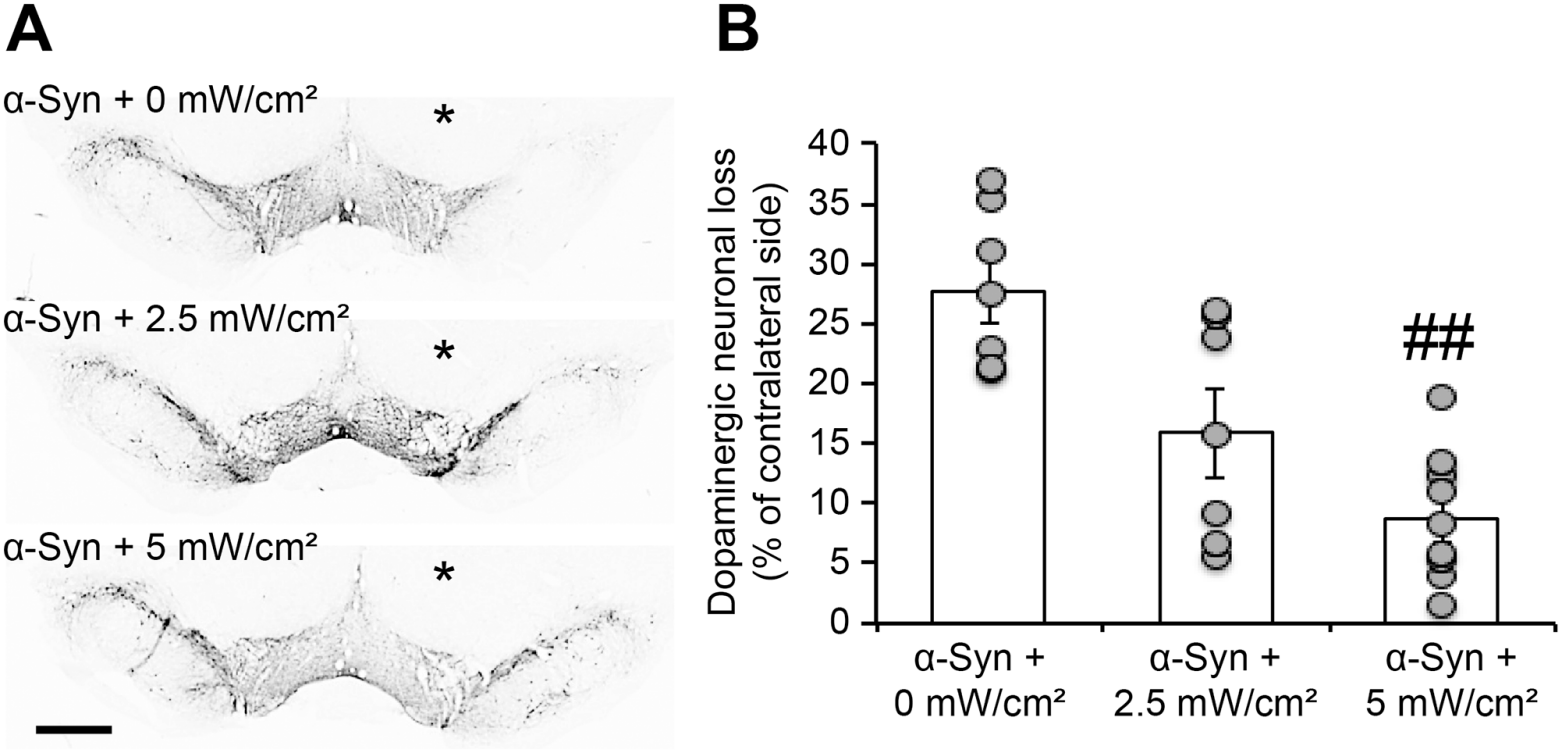
Chronic treatment with photobiomodulation suppresses α-syn-induced dopaminergic neuronal loss. (A) Photomicrographs illustrating dopaminergic (TH+) neuronal loss in the injected substantia nigra. The slices were stained using the anti-TH antibody and the signal revealed a reduction in TH+ immunoreactivity in the injected midbrain (* injected side), compared to the non-injected side. In the treated groups, the immunostaining showed less TH+ signal loss in the injected side, compared to the non-treated animals. Scale bar = 300 μm. (B) Unbiased stereological quantification of TH+ neurons in the injected side (as % of the contralateral side). Comparison with the non-injected side revealed a dramatic neuronal loss in the SNc after human α-syn overexpression (35% of cell loss). Interestingly, comparison of the dopaminergic cell loss in the treated conditions showed less neuronal degeneration, with a significant effect seen only with the fluence rate of 5mW/cm^2^ (## *p*<0.01, Tukey’s test).

We then assessed the integrity of the nigro-striatal dopaminergic system after α-syn overexpression, with and without treatment, by evaluating dopaminergic neuronal loss in the substantia nigra and dopaminergic fiber denervation in the striatum. As we previously reported [19,20], unbiased stereological quantification of the total number of dopaminergic (TH+) neurons in the injected substantia nigra revealed that α-syn overexpression induced significant dopaminergic cell loss (-27% ± 2.7%), four months post-viral delivery (Fig 4). Interestingly, in the treated groups, the rats exhibited less nigral dopaminergic degeneration, with a significant protection against α-syn-induced toxicity observed only after treatment with the higher fluence rate (5mW/cm^2^) (-8.7% ± 1.6%), compared to the control group (*p*<0.01) (Fig 4). Together, these results demonstrate that treatment with PBM could suppress α-syn-induced nigral neuronal degeneration.

**Fig 5:**
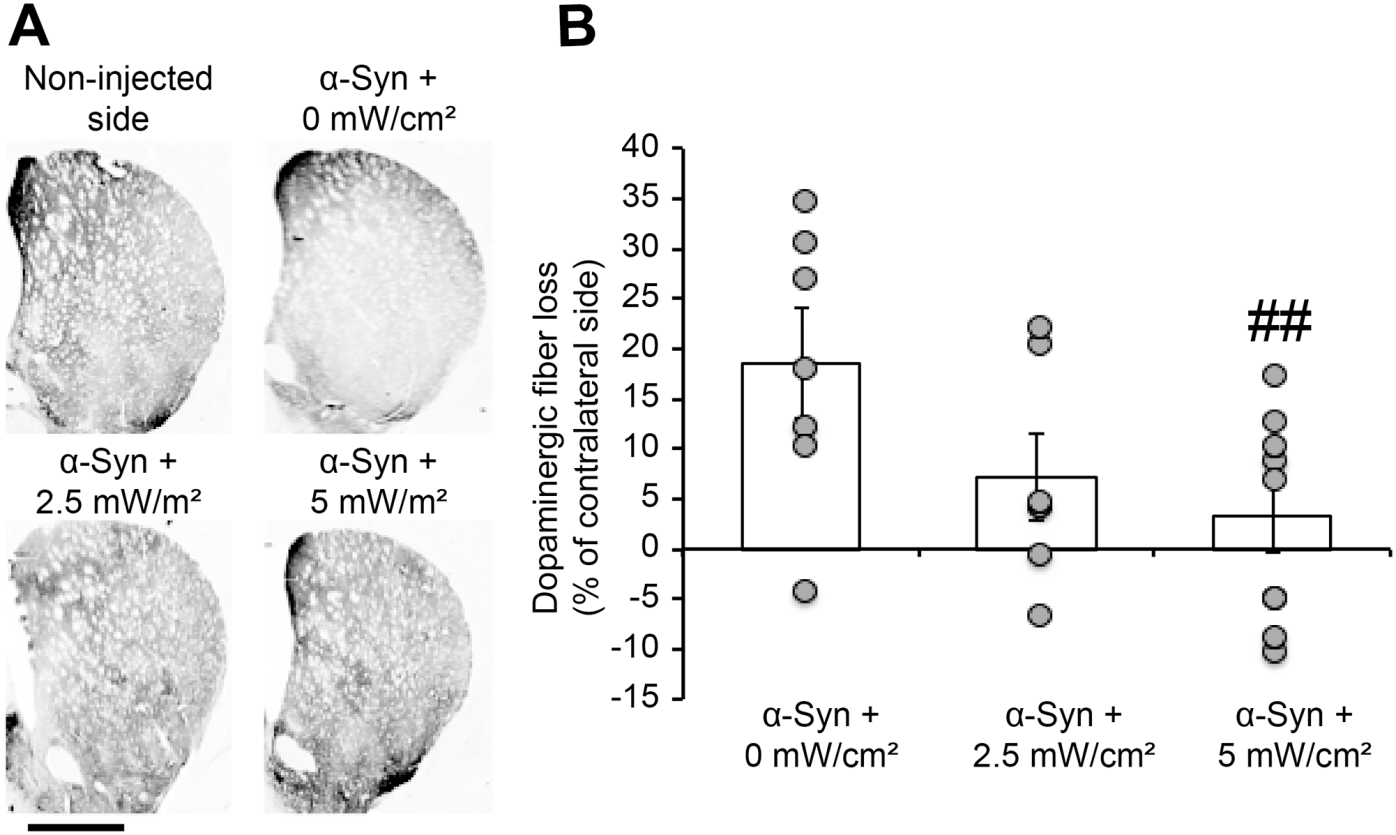
Chronic treatment with photobiomodulation suppresses α-syn-induced dopaminergic fiber denervation in the striatum. (A) Photomicrographs illustrating dopaminergic (TH+) fiber loss in the ipsilateral striatum. The slices were stained using anti-TH antibody and the signal revealed a reduction in TH+ fiber immunoreactivity in the ipsilateral striatum (* injected side). In the treated groups, the immunostaining revealed less TH+ fiber loss in the injected side. Scale bar = 1mm. (B) Optical density quantification of the TH+ dopaminergic fibers in the striatum. Results are expressed as % of TH+ staining signal intensity in the contralateral side. The quantification revealed a dramatic dopaminergic fiber denervation in the ipsilateral striatum. This reduction was not observed after treatment with PBM. A significant effect was only observed with the fluence rate of 5mW/cm^2^ (## *p*<0.01, Tukey’s test).

At the striatal level, quantification of the dopaminergic denervation in the ipsilateral striatum, by measuring optical density of the TH signal, confirmed that human α-syn overexpression induced extensive striatal dopaminergic fiber loss (-18.66% ± 5.6%) (Fig 5), consistent with our previous reports [19,20]. Interestingly, the treated groups exhibited less striatal fiber denervation, with a significant effect observed after treatment at the fluence rate of 5 mW/cm^2^ (-3.21% ± 3.6%), compared to the control group (*p*<0.01) (Fig 5).

### Chronic treatment with photobiomodulation does not affect cell survival in the non-injected midbrain

**Fig 6:**
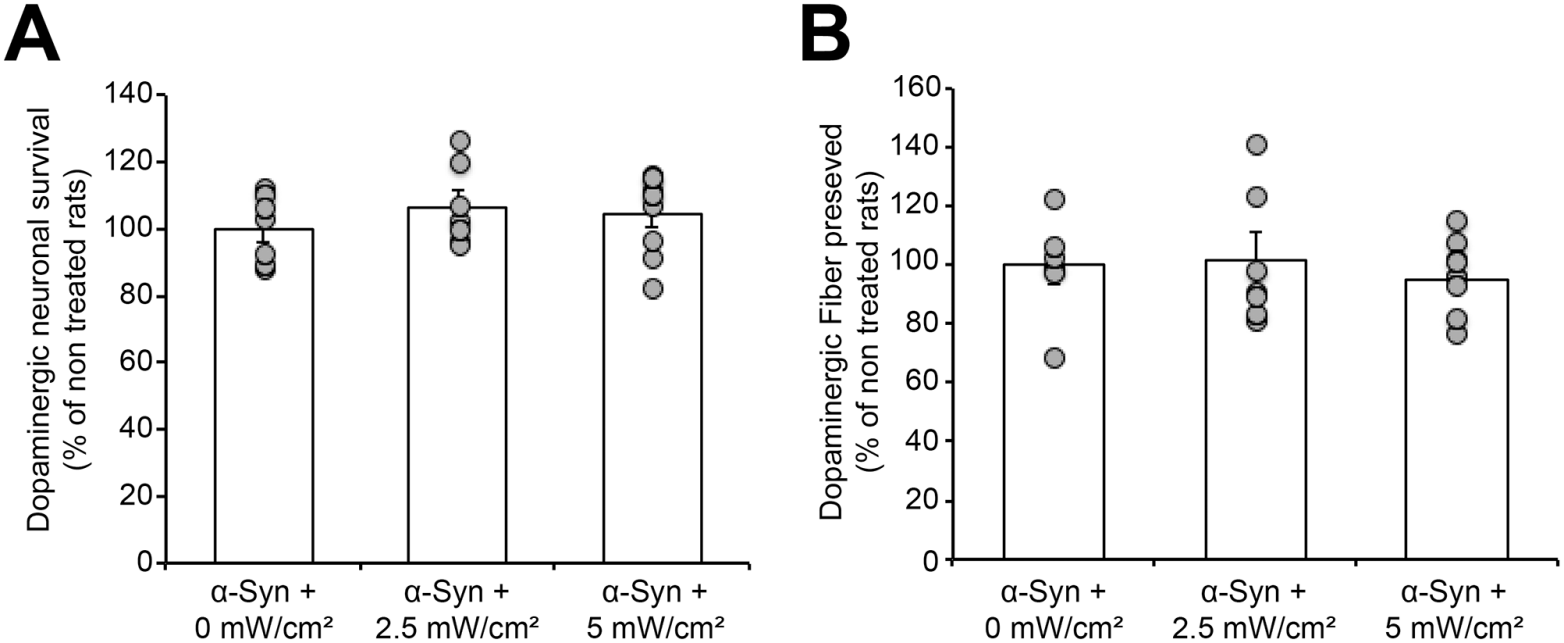
Chronic treatment with photobiomodulation does not affect the integrity of the nigro-striatal dopaminergic pathway in the non-injected side. (A) Unbiased stereological quantification of the total dopaminergic neuronal number in the non-injected side, with or without treatment with PBM, revealed that no difference was observed between all our experimental conditions. (B) Optical density quantification showing that the treated and non-treated animals exhibited similar dopaminergic fiber levels in the contralateral striatum.

To investigate whether treatment with PBM could induce side effects on the healthy neurons, we compared the total number of dopaminergic cells in the non-injected side, with or without exposure to NIR light. Unbiased quantification of TH+ neurons showed that treated and non-treated midbrains exhibited similar total cell numbers, suggesting that chronic treatment with PBM did not affect the survival of non-affected dopaminergic neurons (Fig 6A). At the striatal level, evaluation of the integrity of the dopaminergic fibers showed that the treatment did not induce any significant effect on TH+ fiber density, compared to non-treated animals (Fig 6B).

Moreover, the semi-quantitative evaluation of the total number of cells, using the nuclear counterstain 4',6-diamidino-2-phenylindole, dihydrochloride (DAPI+) in the cortex, the brain region exposed to the highest light fluence rate, revealed that the treatment did not affect cortical cell density (data not shown), confirming that exposure to NIR light by itself, at the fluence rates used in our study, did not damage healthy neurons. It is worth noting that rats exposed to NIR light exhibited a faster post-surgery wound healing and a more rapid hair regrowth compared to non-treated animals, which is consistent with previous reports [29,30]

Together, these results demonstrate that treatment with PMB did not induce any damage to the healthy dopaminergic nigro-striatal pathway or other brain regions, and could have local positive effects on wound healing and hair regrowth.

## Discussion

In the present study, we showed that treatment with PBM suppressed dopaminergic degeneration in a genetic rat model of PD, a well-established model of PD based on the overexpression of human α-syn in the substantia nigra [19,20]. The chronic exposure of rats overexpressing human α-syn to NIR light also mitigated α-syn-induced motor impairment and counteracted the dopaminergic loss in the injected midbrains. Interestingly, we demonstrated that the beneficial effect of PBM persisted several weeks after the last exposure to the NIR light. Furthermore, evaluation of the impact of PBM on the total number of the dopaminergic neurons in the non-injected side revealed that this treatment did not induce any deleterious effects *per se*.

These findings are in line with previous studies that reported a similar positive effect of PBM in a toxin-based animal model of PD. In these studies, the dopaminergic lesion was induced by either acute [15] or chronic [16] administration of 1-methyl-4-phenyl-1,2,3,6-tetrahydropyridine (MPTP) in mice. In both studies, the exposure to NIR light mitigated toxin-induced cell loss and preserved the dopaminergic neurons in the substantia nigra [15,16]. This observation suggests that PBM could act on common cellular pathways activated by overexpression of α-syn or administration of the MPTP toxin.

Moreover, a recent study by Purushothuman and colleagues reported a beneficial effect of PBM on two tau-transgenic models of AD [31]. In these models, exposure to NIR light reduced the levels of pathological hyperphosphorylated tau and protected the neurons against tau toxicity [31]. These findings and the data presented in our study highlight the therapeutic potential of PBM for the treatment of neurodegenerative disorders, including PD and AD, and suggest that PBM might act on common cellular pathways preventing cell death.

It is worth noting that we observed a biphasic response to NIR light on α-syn-induced deleterious effects, with an optimal beneficial effect at the fluence rate of 5 mW/cm^2^ (S1 File). This biphasic response has been previously described in a fly model of PD [18], as well as in other applications of PBM [32,33]. Interestingly, in comparison to other studies, the beneficial fluence rates determined in our animal model is in accordance with the optimal parameters used in other rodent models [15,16,31,34], suggesting that light fluence rates arround 5 mW/cm^2^ could act on common cellular pathways and may represent the therapeutic window.

Although the exact mechanisms underlying the beneficial effect of PBM on several neurodegenerative animal models has yet to be fully elucidated, converging lines of evidence support the hypothesis that this effect could be due to the reduction of mitochondrial dysfunction and/or protection against oxidative stress. In tau transgenic mice and MPTP-treated mice, exposure to NIR light significantly reduced the levels of the oxidative stress markers 4-hydroxynonenal (4-HNE) and 8-hydroxy-2’-deoxyguanosine (8-OHDG) [31,34]. Moreover, several studies showed that treatment with PBM enhances mitochondrial activity and the production of ATP via the direct photo-activation of cytochrome C oxidase, which may play the role of the intracellular photoreceptor [12,18,35–37]. Since α-syn has been reported to induce mitochondrial dysfunction in PD-diseased brains, as well as in PD animal models [38,39], it is plausible that the beneficial effect of PBM in our model could be due in part to the improvement of α-syn-induced deleterious effect on mitochondrial function and reduction of the oxidative stress.

Furthermore, it is also plausible that the beneficial effect of treatment with PBM could be related to a direct impact on α-syn aggregation and toxicity. Indeed, in the APP/PS1 transgenic mice model of AD, Purushothuman and collaborators reported that NIR treatment induced a reduction in the size and number of amyloid-β plaques in the neocortex and hippocampus [31].

### Impact on Parkinson’s disease treatment

The data reported in the current study, in combination with the previous work by Mitrofanis and colleagues [15–17,31,34], point to PBM as a potential therapeutic strategy for the treatment of PD and related synucleinopathies. This strategy presents several advantages: *1)* relatively easy to apply, *2)* sustained beneficial effect several weeks after treatment discontinuation, and *3)* no reported side effects at the optimal doses. Moreover, our data demonstrate that brief and chronic exposure to NIR light induced a beneficial effect when applied during the early stages of the disease, suggesting that this treatment could interfere with the primary events underlying the initiation of PD and related synucleinopathies. Further studies are required to assess the effect of this treatment on other molecular pathways that have been linked to PD pathogenesis, including α-syn aggregation, Lewy bodies formation and pathology spreading.

## Supporting Information

S1 File Supporting information. (PDF)
