# Supplementary material for: Photobiomodulation Suppresses Alpha-Synuclein-Induced Toxicity in an AAV-Based Rat Genetic Model of Parkinson’s Disease
Source: PLoS One. 2015 Oct 20;10(10):e0140880. doi: 10.1371/journal.pone.0140880 (PMC4617694; doi:10.1371/journal.pone.0140880)
Supplement: S1 File — (PDF) [file pone.0140880.s001.pdf]

Determination of the optimal fluence rates of NIR illumination and treatment duration to suppresses α-syn-induced dopaminergic neurodegeneration.

To determine the optimal parameters for the treatment with NIR light (fluence rate and treatment duration), we injected rats with AAV-viral particles to deliver and overexpress human α-syn in the substantia nigra. Three weeks post-injection, the animals were exposed daily, for 100 Sec, to different NIR light (808 nm) fluence rates 5 mW/cm^2 (n=6), 10 mW/cm^2 (n=6), 20 mW/cm^2 (n=6), 25 mW/cm^2 (n=6) and 30 mW/cm^2 (n=6), for the duration of two weeks. The control group (n=5) received AAV injection with sham NIR illumination. During the PBM treatment, awake animals were confined in a transparent Plexiglas cylinder (40 mm ⌀) to limit their movement and to allow the delivery of similar pre-determined light doses to each rat. Animal motor performances were then assessed using the cylinder test before the viral delivery (pre-injection), 2 weeks post-treatment 1 (2 weeks treatment 1) and 14 days after treatment discontinuation (14 days post-treatment 1) (**Figure A**). This test consists of evaluating the use of the contralateral forepaw [1,2], which in the case of α-syn-induced dopaminergic loss, is significantly reduced [3,4]. Analysis showed that, in the control group, overexpression of α-syn induced a motor dysfunction as reflected by the significant reduction of the contralateral forepaw use (**Figure B**, blue histograms), compared to the pre-injection scores (**Figure B**, red histograms). After two weeks of treatment, evaluation of the rat motor performances showed that the rats treated with low fluence rates (5 and 10 mW/cm^2) did not show any significant motor impairment (**Figure B**, blue histograms). However, the rats exposed to higher fluence rates exhibited significant motor impairment, comparable to the non-treated rats (**Figure B**, blue histograms). These results demonstrate that only treatment with low doses of NIR light could mitigate α-syn-induced motor dysfunction.

To evaluate whether the effects of the PBM treatment are sustainable after treatment discontinuation, we assessed rat motor performances two weeks after the last light exposure (**Figure A**). As shown **Figure B** (green histograms, 14 days-post-treatment 1), animals treated with the lowest fluence rates (5 and 10 mW/cm^2) exhibited less motor dysfunction, whereas those treated with higher fluence rates, as well as the non-treated animals, exhibited a significant reduction of the contralateral forepaw use. This observation suggests that the beneficial effect of the PBM is sustainable several days after the last treatment session.

Finally, we submitted the animal to a second session of PBM treatment, for 8 days, and then the rats were sacrificed. Analysis of the rat motor performances after this second session, showed that rats treated with the lowest light fluences rates (5 and 10 mW/cm^2) exhibited less motor dysfunction and those in the other experimental conditions showed a slight motor recovery, probably du to the variability of motor performances within each group (**Figure B**, gray histograms).

At the cellular levels, we first verified the expression of the human α-syn in the injected midbrain. Our results showed that, in all our experimental conditions, the majority of the dopaminergic neurons, in the injected substantia nigra, overexpressed exogenous α-syn (**Figure C**). Unbiased stereological quantification of dopaminergic neurons in the injected midbrain revealed an extensive neuronal loss after human α-syn overexpression, consistent with previous findings from our group [3,4] and others [5]. Interestingly, only low NIR illumination irradiances (5 and 10 mW/cm^2) induced a slight reduction of α-syn-induced neuronal loss, without reaching significant levels

(**Figure D**). In contrast, at higher fluence rates (20, 25 and 30 mW/m2), the rats exhibited similar levels of neuronal loss, compared to the non-treated animals. Moreover, analysis of dopaminergic fiber loss in the ipsilateral striatum confirmed the cellular analysis and revealed a significant loss of dopaminergic fiber density after α-syn overexpression. However, treatment with low fluence rates of NIR illumination suppressed partially the fiber degeneration (**Figure E**). Together, this data demonstrate that the treatment with low fluence rates of NIR illumination may mitigate α-syn-induced motor impairment and dopaminergic neurodegeneration.

References:

1. Kirik D, Georgievska B, Burger C, Winkler C, Muzyczka N, et al. (2002) Reversal of motor impairments in parkinsonian rats by continuous intrastriatal delivery of L-dopa using rAAV-mediated gene transfer. Proc Natl Acad Sci U S A 99: 4708-4713.
2. Schallert T, Fleming SM, Leasure JL, Tillerson JL, Bland ST (2000) CNS plasticity and assessment of forelimb sensorimotor outcome in unilateral rat models of stroke, cortical ablation, parkinsonism and spinal cord injury. Neuropharmacology 39: 777-787.
3. Oueslati A, Paleologou KE, Schneider BL, Aebischer P, Lashuel HA (2012) Mimicking phosphorylation at serine 87 inhibits the aggregation of human alpha-synuclein and protects against its toxicity in a rat model of Parkinson's disease. J Neurosci 32: 1536-1544.
4. Oueslati A, Schneider BL, Aebischer P, Lashuel HA (2013) Polo-like kinase 2 regulates selective autophagic alpha-synuclein clearance and suppresses its toxicity in vivo. Proc Natl Acad Sci U S A 110: E3945-3954.
5. Azeredo da Silveira S, Schneider BL, Cifuentes-Diaz C, Sage D, Abbas-Terki T, et al. (2009) Phosphorylation does not prompt, nor prevent, the formation of alpha-synuclein toxic species in a rat model of Parkinson's disease. Hum Mol Genet 18: 872-887.

fluence rate ***at the midbrain*** ***(mW/cm²)***	***Irradiance at scalp surface*** ***(mW/cm²)***
2.5	20.4
5	40.8
10	81.6
20	161.1
25	204
30	244.8

Table A. Assessment of the NIR light fluence rates in rat midbrain. A light detector was introduced in a freshly decapitated rat head through the spinal cord tract to reach deep brain regions, including the midbrain.

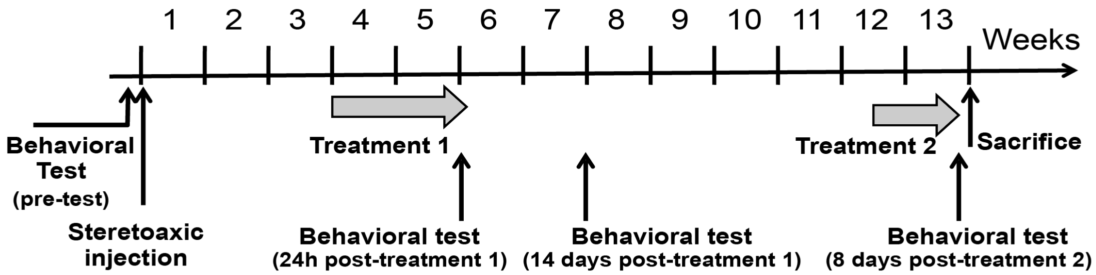

Figure A. Experimental design. Schematic representation of the experimental timeline showing the two periods of treatment with PBM and the different behavioral test sessions.

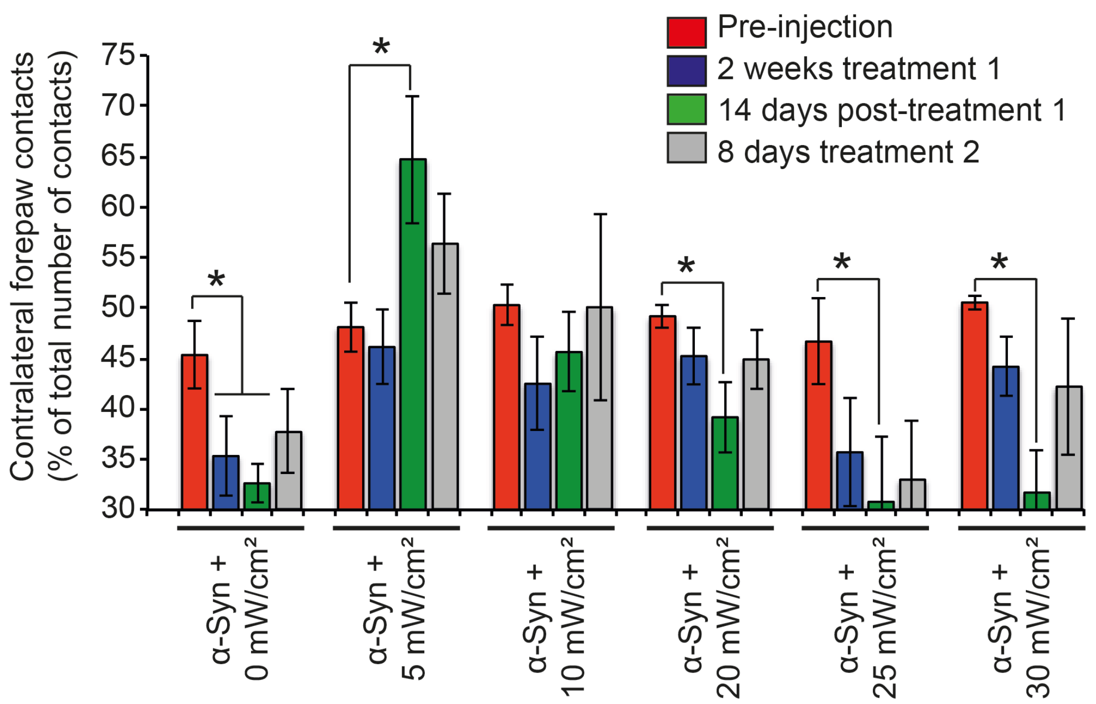

Figure B. Effect of chronic treatment with photobiomodulation on α-syn-induced motor deficits. α-syn-induced motor deficits were assessed using the cylinder test. The results showed that, before the injection (red histograms), animals in all our experimental conditions exhibited symmetrical motor activity, with 50% of use for ipsilateral and contralateral forepaws. In the non treated group, α-syn overexpression induced a significant reduction of the use of the contralateral forepaw (*$p<0.05$ *vs.* pre-injection scores, Bonferroni's test). In the NIR treated groups, while animals exposed to low fluence rates (5 and 10 mW/cm^2) did not show visible motor dysfunctions, rats treated with high fluence rates (20, 25 and 30 mW/cm^2) exhibited significant reduction of the contralateral forepaw use (*$p<0.05$ *vs.* pre-injection scores, Bonferroni's test).

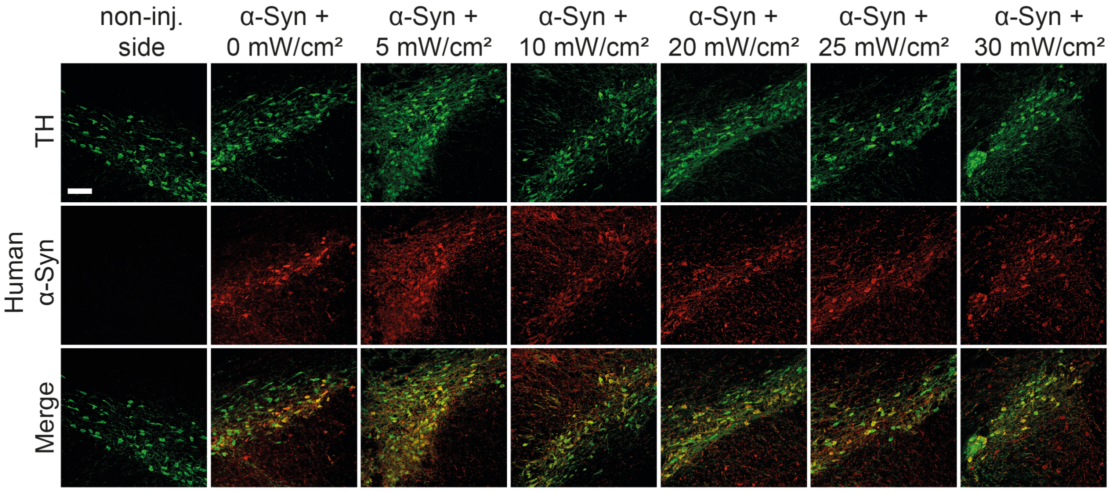

Figure C. Expression of human α-syn in the injected substantia nigra. Photomicrographs illustrating the detection of human α-syn expression (red) in nigral TH+ dopaminergic neurons (green). The merge shows that human α-syn localizes in the majority of the dopaminergic neurons in the injected midbrain. No human α-syn signal was observed in the non-injected side. Scale bar = 500 μm.

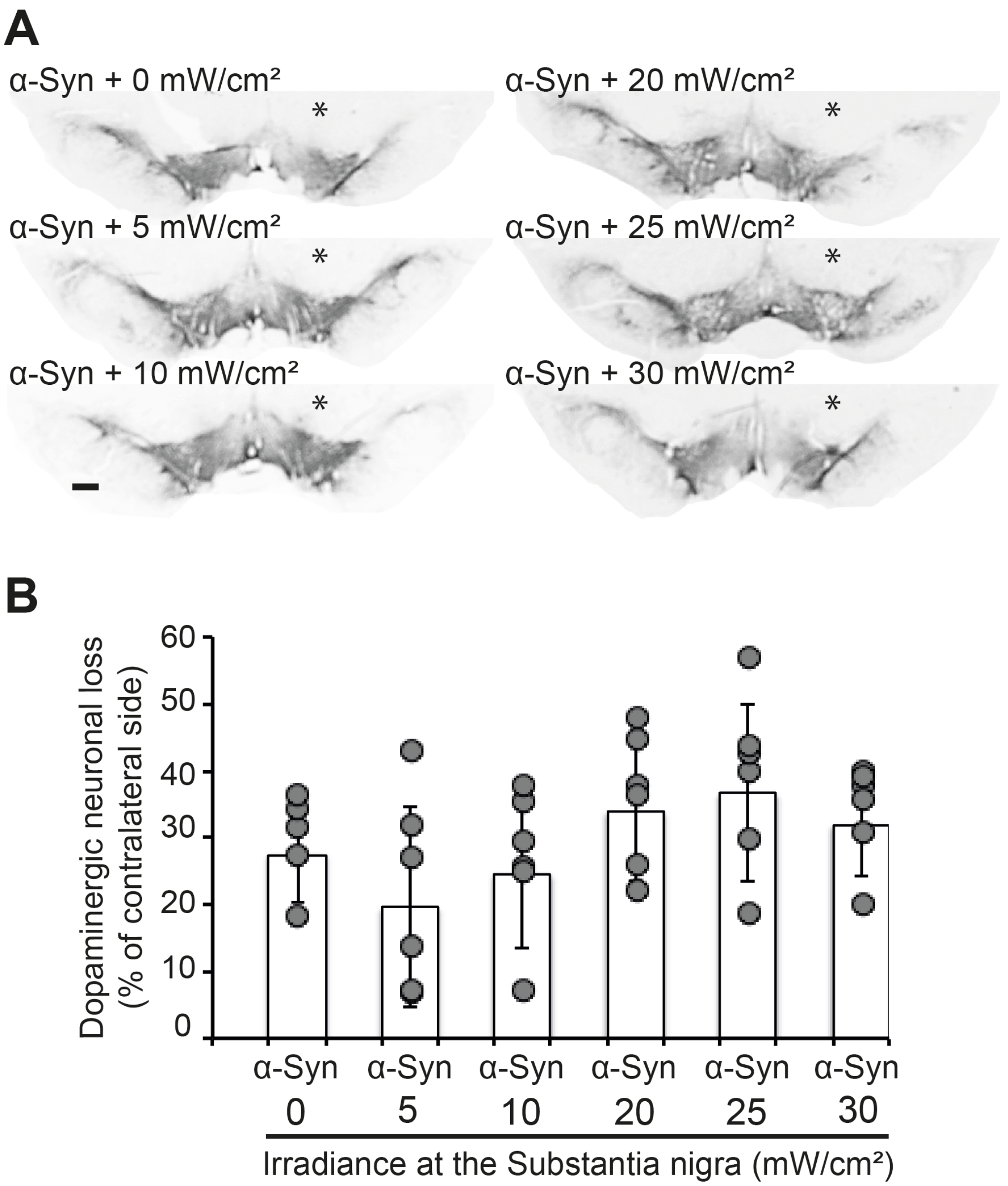

Figure D. Effect of chronic treatment with photo-biomobulation on α-syn-induced dopaminergic neuronal loss. (A) Photomicrographs illustrating dopaminergic (TH+) neuronal loss in the injected substantia nigra. Slices were stained using anti-TH antibody and the signal revealed a reduction in TH+ immunoreactivity in the injected midbrain (* injected side), compared to the non-injected side. In the treated groups, the immunostaining did not show any clear effect α-syn induced dopaminergic cell loss. Scale bar = 300 µm. (B) Unbiased stereological quantification of TH+ neurons in the injected side (as % of the contralateral side). Comparison with the non-injected side revealed a significant neuronal loss in the SNc after human α-syn overexpression. In the treated groups, exposure to the NIR light did not affect α-syn-induced cell loss. However, only the lowest fluence rates showed a tendency to reduce α-syn toxicity, without reaching a significant level.

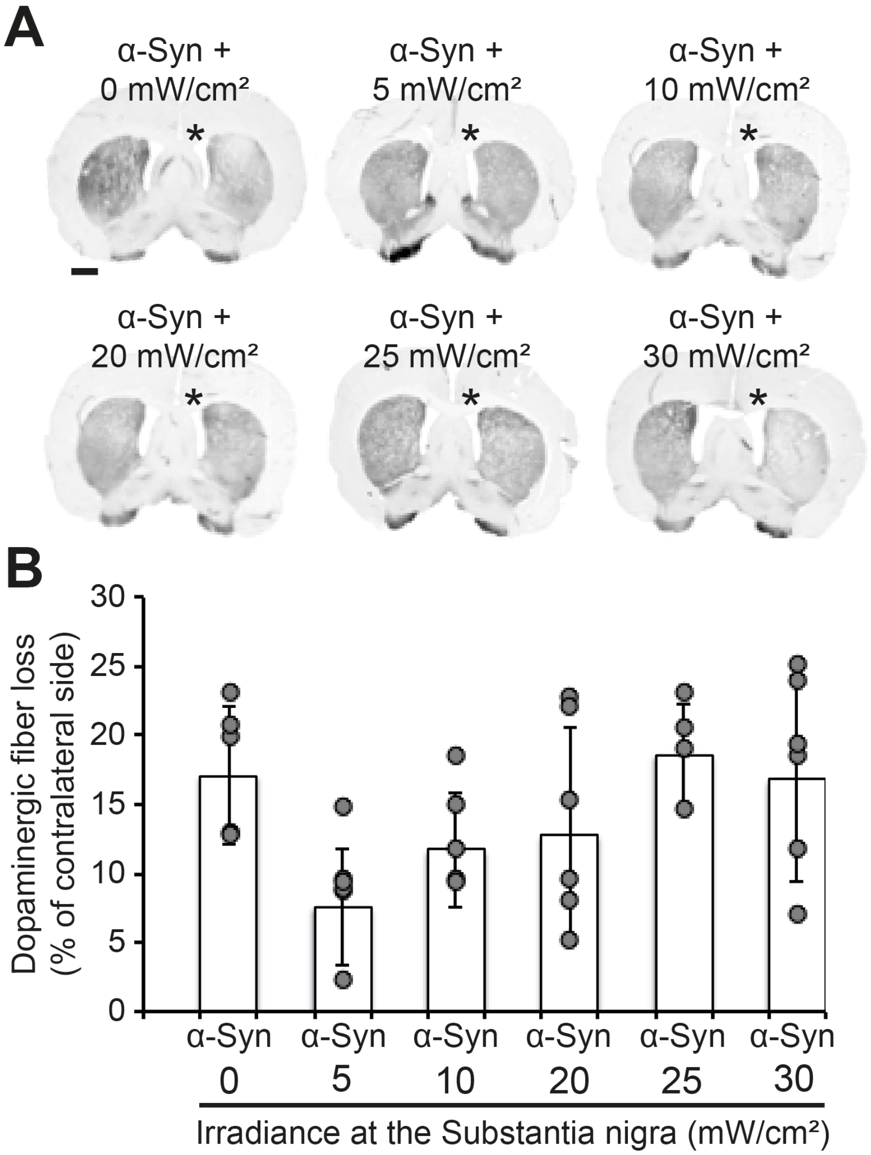

Figure E. Effect of chronic treatment with photo-biomobulation on α-syn-induced dopaminergic fiber denervation in the striatum. (A) Photomicrographs illustrating dopaminergic (TH+) fiber loss in the ipsilateral striatum. Slices were stained using anti-TH antibody and the signal revealed a reduction in TH+ fiber immunoreactivity in the ipsilateral striatum (* injected side). In the treated groups, the immunostaining revealed less TH+ fiber loss in the injected side. Scale bar = 1mm. (B) Optical density quantification of TH+ dopaminergic fibers in the striatum. Results are expressed as % of the signal TH+ staining intensity in the contralateral side. The quantification revealed a significant dopaminergic fiber denervation in the ipsilateral striatum. This reduction was partially mitigated by the treatment with the lowest NIR fluence rate (5mW/cm^2).
